# Understanding Concerns, Sentiments, and Disparities Among Population Groups During the COVID-19 Pandemic Via Twitter Data Mining: Large-scale Cross-sectional Study

**DOI:** 10.2196/26482

**Published:** 2021-03-05

**Authors:** Chunyan Zhang, Songhua Xu, Zongfang Li, Shunxu Hu

**Affiliations:** 1 Institute of Medical Artificial Intelligence The Second Affiliate Hospital of Xi’an Jiaotong University Xi'an China; 2 School of Mathematics and Statistics Xi’an Jiaotong University Xi'an China

**Keywords:** COVID-19, Twitter mining, infodemiology, infoveillance, pandemic, concerns, sentiments, population groups, disparities

## Abstract

**Background:**

Since the beginning of the COVID-19 pandemic in late 2019, its far-reaching impacts have been witnessed globally across all aspects of human life, such as health, economy, politics, and education. Such widely penetrating impacts cast significant and profound burdens on all population groups, incurring varied concerns and sentiments among them.

**Objective:**

This study aims to identify the concerns, sentiments, and disparities of various population groups during the COVID-19 pandemic through a cross-sectional study conducted via large-scale Twitter data mining infoveillance.

**Methods:**

This study consisted of three steps: first, tweets posted during the pandemic were collected and preprocessed on a large scale; second, the key population attributes, concerns, sentiments, and emotions were extracted via a collection of natural language processing procedures; third, multiple analyses were conducted to reveal concerns, sentiments, and disparities among population groups during the pandemic. Overall, this study implemented a quick, effective, and economical approach for analyzing population-level disparities during a public health event. The source code developed in this study was released for free public use at GitHub.

**Results:**

A total of 1,015,655 original English tweets posted from August 7 to 12, 2020, were acquired and analyzed to obtain the following results. Organizations were significantly more concerned about COVID-19 (odds ratio [OR] 3.48, 95% CI 3.39-3.58) and expressed more fear and depression emotions than individuals. Females were less concerned about COVID-19 (OR 0.73, 95% CI 0.71-0.75) and expressed less fear and depression emotions than males. Among all age groups (ie, ≤18, 19-29, 30-39, and ≥40 years of age), the attention ORs of COVID-19 fear and depression increased significantly with age. It is worth noting that not all females paid less attention to COVID-19 than males. In the age group of 40 years or older, females were more concerned than males, especially regarding the economic and education topics. In addition, males 40 years or older and 18 years or younger were the least positive. Lastly, in all sentiment analyses, the sentiment polarities regarding political topics were always the lowest among the five topics of concern across all population groups.

**Conclusions:**

Through large-scale Twitter data mining, this study revealed that meaningful differences regarding concerns and sentiments about COVID-19-related topics existed among population groups during the study period. Therefore, specialized and varied attention and support are needed for different population groups. In addition, the efficient analysis method implemented by our publicly released code can be utilized to dynamically track the evolution of each population group during the pandemic or any other major event for better informed public health research and interventions.

## Introduction

### Background

Since December 2019, COVID-19 has rapidly spread all over the world and caused millions of deaths [1,2]. Although many countries have implemented various countermeasures [3,4], an end to the pandemic is still not in sight. So far, COVID-19 has already exerted tremendous impacts across various aspects of human life, such as health, economy, politics, and education [5-8], whose influences may last for an unknown period. Such widely penetrating and long-lasting impacts are likely to cause disproportionate burdens on different population groups, incurring varied concerns and sentiments among them. Therefore, it is of great importance to understand the disparities in the responses of these population groups to COVID-19 for better informed public health research and intervention.

### Literature Reviews

So far, two classes of methods have been utilized to study the impacts of COVID-19 on public and personal life, including large-scale social media mining approaches and cross-sectional analyses through online and offline questionnaires, which are briefly reviewed in the following text.

The first class of methods provides a fast and economical way to analyze the population impacts of COVID-19 through mining social media data generated during the pandemic. Currently, such methods have been employed in a number of studies. For example, Lwin et al [9] studied Twitter data to explore global trends of four emotions—fear, anger, sadness, and joy—as well as their relative salience. After studying the topics obtained by latent Dirichlet allocation topic modeling on Twitter text data, Abd-Alrazaq et al [10] identified the sentiments of four major topics and 12 subtopics, and showed that all topics were positive except for two (ie, death and racial discrimination). Similarly, Hung et al [11] adopted the Valence Aware Dictionary and Emotional Reasoner (VADER) model to analyze the sentiments expressed in user tweets and found that positive, neutral, and negative emotions accounted for 48.2%, 20.7%, and 31.1% of the tweets, respectively.

Despite the informative understanding regarding people’s sentiments provided by these prior studies, it is noted that these existing methods tend to treat their study population as a whole in the analysis, ignoring likely disparities among population groups. Case reports from many countries and epidemiological research on COVID-19 state that the morbidity and mortality of COVID-19 are related to age and gender [12-14], calling for a more fine-grained analysis regarding the concerns and sentiments of each population group during the pandemic.

The second class of methods has been popularly leveraged to understand the health statuses of population groups, uncover health-related factors, and carry out disease epidemiology research. Table 1 [15-20] lists some representative cross-sectional surveys on COVID-19. Compared with the first class of data mining methods, cross-sectional studies can provide richer and more fine-grained information through well-controlled questionnaires, which is of great use for analyzing the detailed disparities of population groups.

**Table 1:** Representative cross-sectional studies on COVID-19.

Author and reference	Study target area	Study period (all in 2020)	No. of participants (online or offline)	Highlights
Liu et al [15]	Wuhan and surrounding cities, China	January 30-February 8	300 (online)	Gender differences exist in posttraumatic stress symptoms during COVID-19: females suffer more than males.
Lu et al [16]	Fujian, China	April 6-22	2299 (offline)	Work differences exist in fear, anxiety, and depression emotions in hospitals during COVID-19: medical workers suffer more than administrative workers.
Nelson et al [17]	Parts of the United States	March 14-16	9009 (online)	Age differences exist in concerns about COVID-19: people aged 40-54 years and 55-75 years are very worried and extremely worried population groups, respectively.
Groarke et al [18]	The United Kingdom	March 23-April 24	1964 (online)	Age differences exist in loneliness during COVID-19: young people suffer most.
Azlan et al [19]	Malaysia	March 27-April 3	4850 (online)	Gender, age, region, occupation, and income differences exist in public knowledge toward COVID-19.
Ahmad and Murad [20]	Iraqi Kurdistan	Not stated	516 (online)	Age differences exist in mental health during COVID-19: young people aged 18-35 years are facing psychological anxiety.

However, the shortcomings of both online and offline cross-sectional studies are also commonly acknowledged. In particular, launching offline questionnaires during the COVID-19 pandemic may pose eminent public health hazards because of the risk of virus transmission through personal contacts. Online questionnaires also have their own challenges, mainly difficulties in finding an adequate number of willing participants to complete the online questionnaires honestly and at a high quality. The operational obstacle of online questionnaires is further elevated if repeated surveys are intended to track the dynamic evolution of population groups regarding their thoughts and needs [21].

Recognizing the limitations of the two classes of existing study methods, in this work, we conducted a new cross-sectional study via large-scale Twitter data mining. Through this method, we aimed to identify the concerns, sentiments, and disparities of various population groups during the COVID-19 pandemic in fine granularity without administrating any online or offline questionnaires. The advantage of our approach lies in its economic and efficient way of gathering multifaceted awareness information from population groups and their disparities. With such an understanding of the concerns and sentiments of population groups regarding COVID-19, specialized attention and customized programs can be developed to assist each population group. It is noted that the method implemented through our social media data mining approach can be easily repurposed to study the evolution of different population groups during any major public health event for better informed public health research and interventions. The source code developed in this study has been released for free public use at GitHub [22].

## Methods

**Figure 1:**
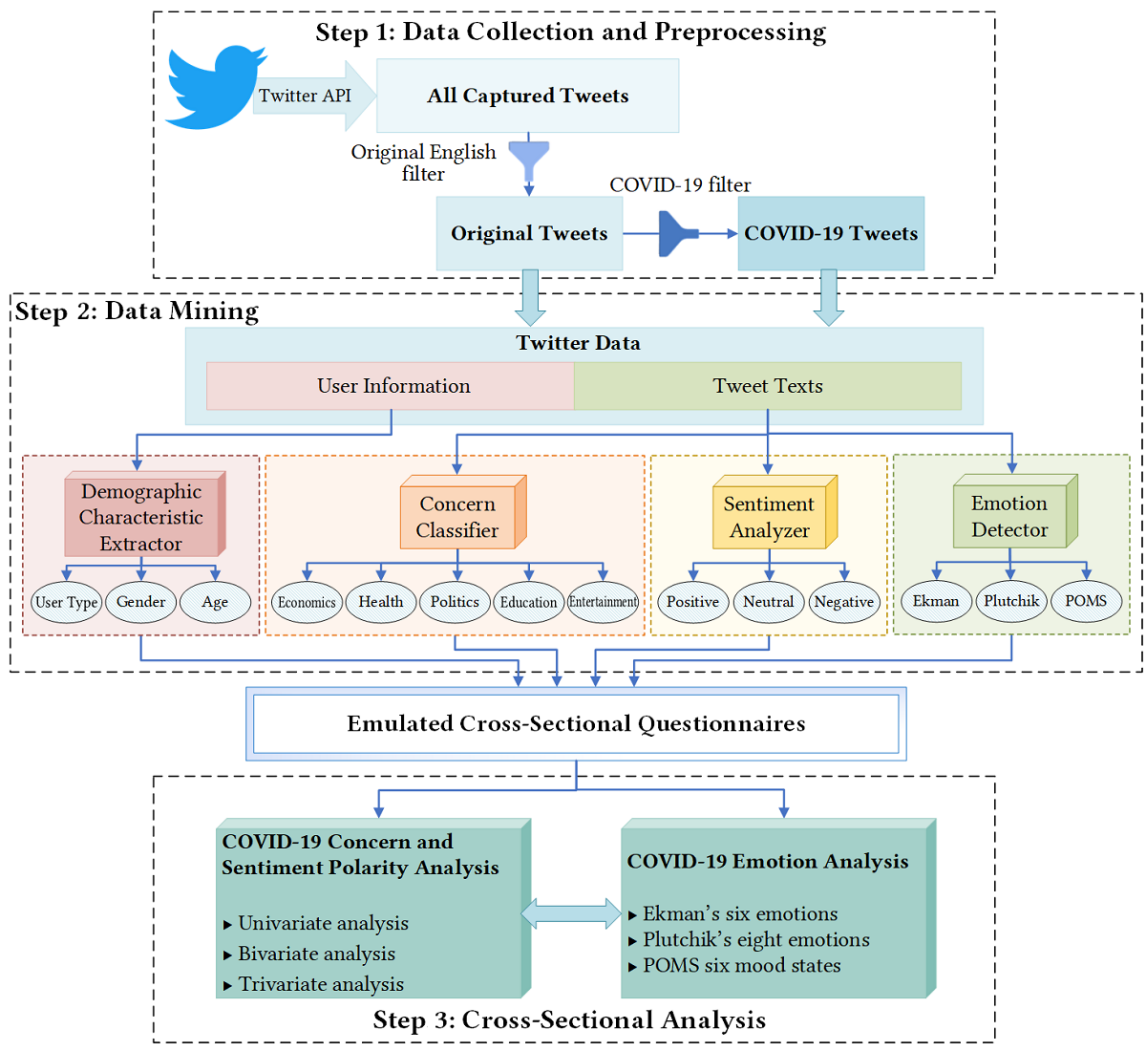
The structure of our cross-sectional method. API: application programming interface; POMS: Profile of Mood States.

As shown in Figure 1, the cross-sectional method proposed in this study consists of three steps. The implementation details of each step are described in the following sections.

### Data Collection and Preprocessing

The Twitter data used in this study were collected by sampled stream application programming interface v1 [23] and v2 [24] from Twitter Developer Labs, which can stream about 1% of publicly available tweets in real time. Meanwhile, detailed author data from all the tweets were collected to extract population characteristics. Unlike those in other research studies on Twitter [9-11], the data captured in this study are a random sampling of all Twitter data without using any filter, which can better reflect the common opinions in people’s daily lives. As of November 2020, we have collected, in total, more than 600 million tweets (ie, over 2 Terabytes) during the COVID-19 pandemic.

**Figure 2:**
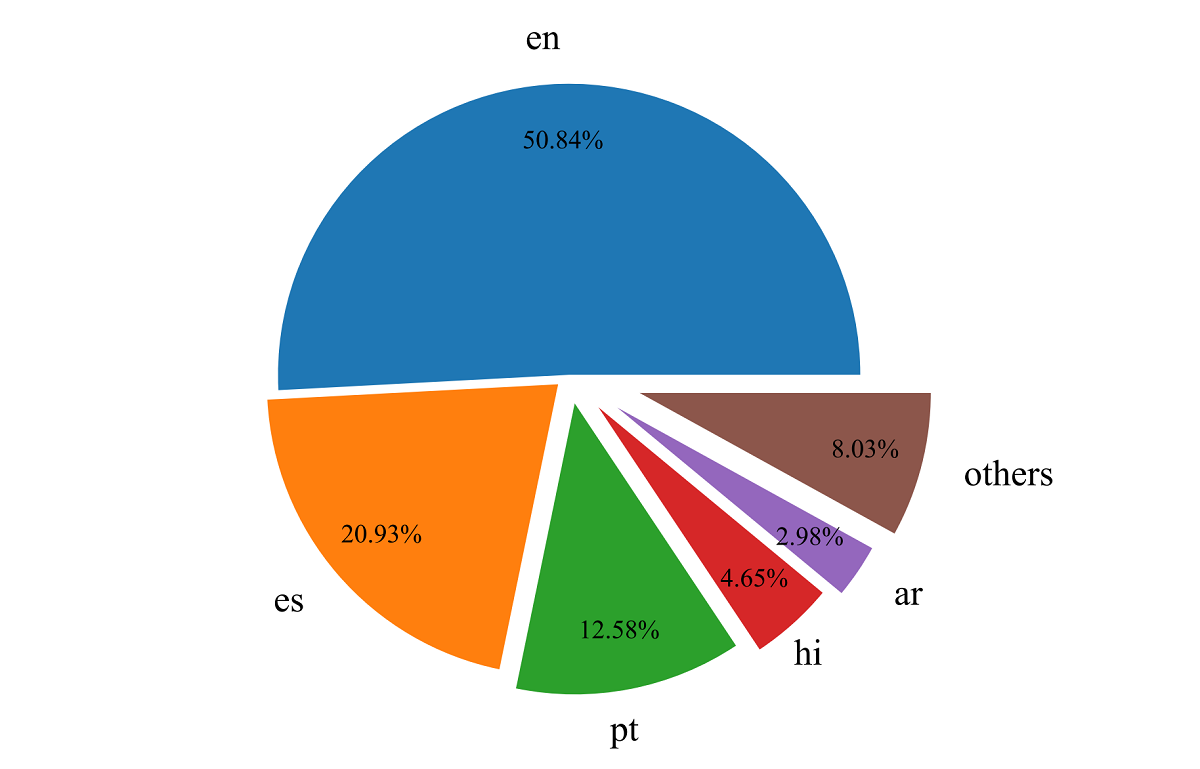
The language distribution of tweets. ar: Arabic; en: English; es: Spanish; hi: Hindi; others: other languages; pt: Portuguese.

In the data preprocessing step, an original English filter and a COVID-19 filter were used to generate the original and COVID-19 tweet data sets based on all the captured tweets. Since original tweets can better reflect the authors’ dynamic thoughts and sentiments, and English tweets comprise over half of all tweets (see Figure 2), we only focused on original English tweets, which can be filtered by the attributes of the tweet object. In order to obtain COVID-19 tweets, we made a filter pattern that is composed of 590 COVID-19 keywords and hashtags provided by Twitter COVID-19 filter rules [25].

### Data Mining

Data mining is the key step in emulating cross-sectional questionnaires based on the two tweet data sets. This step contained four intelligent modules: demographic characteristic extractor, concern classifier, sentiment analyzer, and emotion detector.

#### Demographic Characteristic Extractor

**Figure 3:**
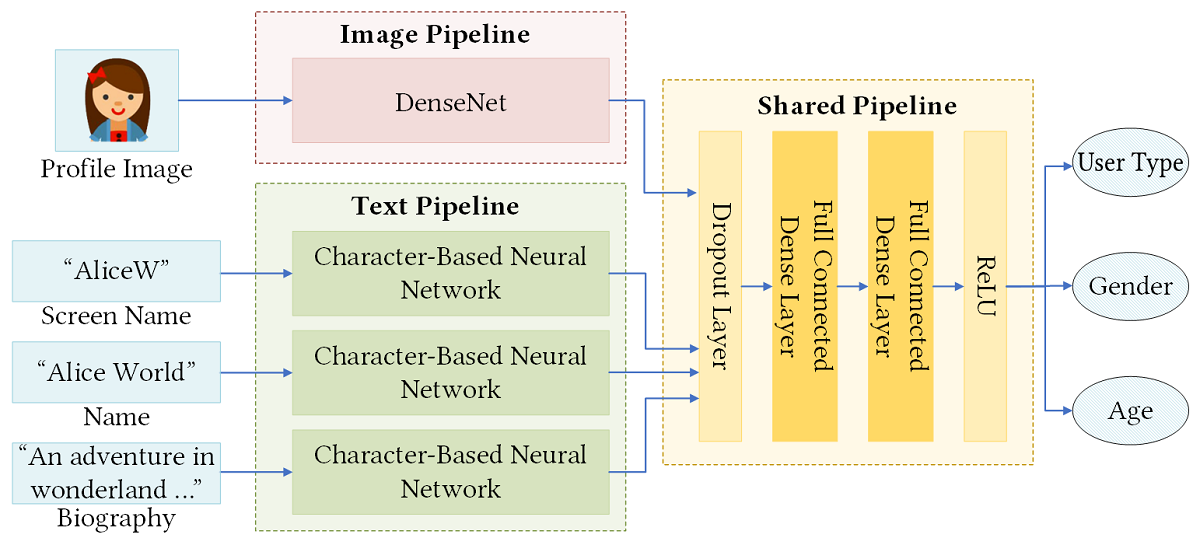
The structure of the M3 (multimodal, multilingual, and multi-attribute) model for inferring user type, gender, and age from profile information. DenseNet: dense convolutional network; ReLU: rectified linear unit.

This module was used to extract three demographic characteristics—user type, gender, and age—through profile images, screen names, names, and biographies. It was implemented by an open source package of the M3 (multimodal, multilingual, and multi-attribute) model [26], which is a multimodal deep neural system trained on a massive data set, composed of Twitter, IMDB, and Wikipedia data [27], for demographic inference. In this M3 model, user type (ie, person or organization) and gender (ie, male or female) were modeled as binary classification tasks, while age was modeled as a 4-class classification task with the following age groups: ≤18, 19-29, 30-39, and ≥40 years of age. As shown in Figure 3, the structure of the M3 model consisted of two separate pipelines—image pipeline and text pipeline—and a shared pipeline. The image pipeline was employed to process profile images using the dense convolutional network (DenseNet) [28], and the text pipeline was used for processing three text sources of screen names, names, and biographies by adopting three character-based neural networks. The shared pipeline combined the outputs of the two separate pipelines and then mainly applied two fully connected dense layers to predict the user type, gender, and age state of each Twitter user. All of these pipelines were fine-tuned to capture accurate demographic features. For more detailed information, readers can refer to the original literature [26].

We tested the M3 model on a subset of our original English tweets that carried ground-truth labels of user type, gender, and age explicitly or implicitly; the detection procedure is explained in detail in Multimedia Appendix 1. The benchmark performance of the M3 model on this subset is as follows: for user type, gender, and age, the accuracy scores are 99.07%, 95.88%, and 77.65%, respectively, and the macro–F1 scores are 0.9860, 0.9572, and 0.7311, respectively.

#### Concern Classifier

This module was used to classify the tweets into five categories of human life—economics, politics, health, education, and entertainment—which was based on our self-designed matching patterns. First, five specialized vocabulary dictionaries were collected and constructed from Oxford Reference and other sources, including an economic vocabulary (ie, A Dictionary of Economics [29] and The Economist [30]) and a political vocabulary (ie, A Concise Oxford Dictionary of Politics and International Relations [31]). Then, the vocabulary dictionaries were imported into the matching patterns in a regular expression format, with which we labeled all the tweets.

#### Sentiment Analyzer

This module calculated the sentiment polarities of the tweets based on the VADER [32] model. The VADER model is a sentiment analysis tool based on lexicons of sentiment-related words, which can automatically classify each word in the lexicon as positive, neutral, or negative. The range of the sentiment polarity is –1 to 1, which is divided into three subranges: negative (–1 to –0.05), neutral (–0.05 to 0.05), and positive (0.05 to 1).

#### Emotion Detector

This module is based on an emotion recognition model on Twitter [33], which utilizes a character-based trained recurrent neural network algorithm. It employs three emotion models to recognize different human emotions, including Ekman’s six basic emotions model [34]; Plutchik’s eight primary emotions model, also known as the emotion wheel [35]; and the Profile of Mood States (POMS) model [36], which measures six mood states. Based on the above-mentioned modules, the template of the emulated cross-sectional questionnaire is shown in Table 2.

**Table 2:** The template of the cross-sectional questionnaire.

Question category		Response categories
**Population characteristic**		
	User type	Person Organization
	Gender	Male Female
	Age (years)	≤18 19-29 30-39 ≥40
**Concern**		
	Economics	Concerned Unconcerned
	Health	Concerned Unconcerned
	Politics	Concerned Unconcerned
	Education	Concerned Unconcerned
	Entertainment	Concerned Unconcerned
**Sentiment polarity**		
	Negative	–1 to –0.05
	Neutral	–0.05 to 0.05
	Positive	0.05 to 1
**Emotions**		
	Ekman’s six emotions: anger, disgust, fear, joy, sadness, and surprise	0 to 1 for each emotion
	Plutchik’s eight emotions: anger, disgust, fear, joy, sadness, surprise, trust, and anticipation	0 to 1 for each emotion
	POMS^a^ six emotions: anger, depression, fatigue, vigor, tension, and confusion	0 to 1 for each emotion

^a^POMS: Profile of Mood States.

### Cross-sectional Analysis

The purpose of this step was to analyze the concerns and sentiments of different population groups in response to COVID-19 based on the Twitter data mining outcomes of the emulated questionnaire. It includes two parts: the COVID-19 concern and sentiment polarity analysis and the COVID-19 emotion analysis. The odds ratio (OR) was employed in these two parts to compare the relative ratios of population groups under multiple variable conditions. Meanwhile, we used the chi-square test to measure the significance level of difference (ie, *P* value) under each condition.

## Results

### Overall Analysis

During the COVID-19 pandemic, various emotions were expressed by the general public. To study the disparities between different population groups during this period, we conducted a cross-sectional analysis on the daily Twitter data collected from August 7 to 12, 2020. In total, 7,590,844 unfiltered tweets were captured during the research period, of which 1,015,655 were original English tweets; these are referred to as the original data set. From this original data set, 27,216 tweets were related to COVID-19; these are referred to as COVID-19 data set. The statistical distributions and *P* values, by chi-square test, of the two data sets are shown in Table 3.

We can see from Table 3 that the population groups under each variable all showed significant differences (*P*<.001) in response to COVID-19. As shown in Table 3, 89.94% of the total participants were persons and 10.06% were organizations. As a comparison, 73.00% and 27.00% of COVID-19-related participants were persons and organizations, respectively. The total proportion of male participants on social media was slightly higher than that of females (52.74% vs 47.26%), while this gap was further widened to 60.38% versus 39.62% under COVID-19, respectively. The total proportions of the four age groups—≤18, 19-29, 30-39, and ≥40 years of age—were 37.93%, 38.42%, 11.41%, and 12.24%, respectively; from this, it can be inferred that people below 30 years of age are more active on social media. Under COVID-19, the proportions increased in the age groups above 30 years and decreased in the age groups below 30 years; thus, the proportions of the four age groups changed to 17.83%, 29.18%, 18.32%, and 34.67%, respectively. The total proportions of the five topics—economics, health, politics, education, and entertainment—were 13.99%, 13.90%, 7.27%, 6.38%, and 7.79%, respectively; under COVID-19, their proportions changed to 34.30%, 22.60%, 19.97%, 15.74%, and 6.38%, respectively. The total proportions of positive, neutral, and negative sentiments were 42.46%, 31.38%, and 26.16%, respectively; the mean sentiment polarity was 0.1067 (SD 0.4647). Under COVID-19, the proportions of positive, neutral, and negative sentiments were 43.15%, 24.37%, and 32.48%, respectively; the mean sentiment polarity fell to 0.0659 (SD 0.4941).

**Table 3:** Statistical distributions of the emulated questionnaire answers.

Variable		Total tweets, n (%)^a^	COVID-19-related tweets, n (%)	*P* value
Overall		1,015,655 (100)	27,216 (100)	N/A^b^
**User type**				
	Person	913,480 (89.94)	19,869 (73.00)	<.001
	Organization	102,175 (10.06)	7347 (27.00)	N/A
**Gender**				
	Male	481,770 (52.74)	11,997 (60.38)	<.001
	Female	431,710 (47.26)	7872 (39.62)	N/A
**Age (years)**				
	≤18	346,483 (37.93)	3542 (17.83)	<.001
	19-29	350,959 (38.42)	5798 (29.18)	N/A
	30-39	104,228 (11.41)	3640 (18.32)	N/A
	≥40	111,810 (12.24)	6889 (34.67)	N/A
**Concern**				
	Economics	142,090 (13.99)	9334 (34.30)	<.001
	Health	141,176 (13.90)	6152 (22.60)	N/A
	Politics	73,838 (7.27)	5434 (19.97)	N/A
	Education	64,799 (6.38)	4284 (15.74)	N/A
	Entertainment	79,119 (7.79)	1736 (6.38)	N/A
**Sentiment polarity**				
	Overall (–1 to 1), mean (SD)	0.1067 (0.4647)	0.0659 (0.4941)	<.001
	Positive (–1 to 0.05)	431,247 (42.46)	11,744 (43.15)	<.001
	Neutral (–0.05 to 0.05)	318,713 (31.38)	6632 (24.37)	N/A
	Negative (0.05 to 1)	265,695 (26.16)	8840 (32.48)	N/A

^a^All values are expressed as n (%), except for overall sentiment polarity, which is expressed as mean (SD).

^b^*P* values were calculated for the main variables and not for individual responses.

The above analysis cannot provide fine-grained differences between population groups under multivariate conditions. To understand these differences more clearly, we adopted a cross-sectional analysis based on the emulated questionnaire outcomes, which consists of two parts: one is COVID-19 concern and sentiment polarity analysis, including univariate, bivariate, and trivariate analysis, and the other one is COVID-19 emotion analysis, including three emotion models. The analysis process and results are presented in the following sections.

### COVID-19 Concern and Sentiment Polarity Analysis

#### Univariate Analysis

**Figure 4:**
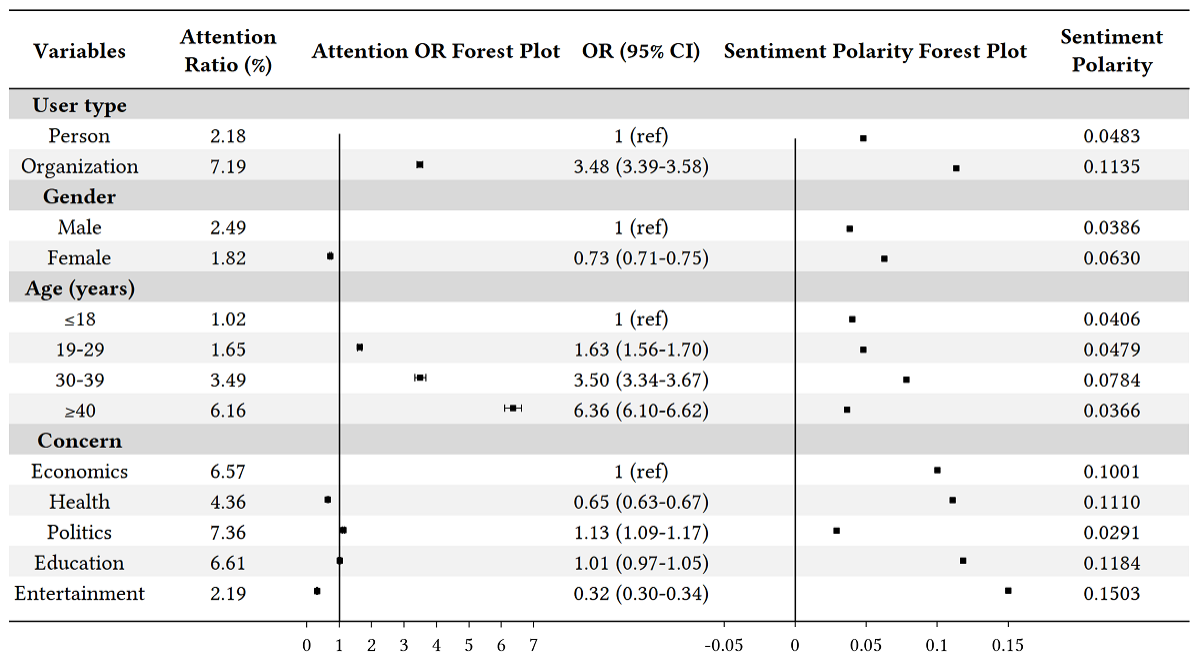
Univariate analysis of COVID-19 concerns and sentiment polarities among different population groups. OR: odds ratio.

The population characteristics in this study included four variables—user type, gender, age, and concern—on which we first performed a univariate statistical analysis of COVID-19 concerns and sentiment polarities. The results are shown in Figure 4.

It can be seen that the organizations’ attention ratio (7.19%) to COVID-19 was significantly higher than that of individuals (2.18%), and the attention OR of organizations was 3.48 (95% CI 3.39-3.58) compared with individuals. Moreover, organizations’ sentiment polarity (0.1135) was more positive than that of individuals (0.0483). The COVID-19 attention ratio of females (1.82%) was a bit lower than that of males (2.49%), with an attention OR of 0.73 (95% CI 0.71-0.75). Meanwhile, females were more positive than males, and the sentiment polarities were 0.0630 and 0.0386 for females and males, respectively. In addition, COVID-19 attention increased significantly with age. Among the four age groups, the attention ORs of the groups that were 19 to 29 years, 30 to 39 years , and 40 years or older were 1.63 (95% CI 1.56-1.70), 3.50 (95% CI 3.34-3.67) and 6.36 (95% CI 6.10-6.62), respectively, in comparison with the group that was 18 years or less, which implies that older people are more concerned about COVID-19. The group that was 40 years or older was less positive than other age groups, with a sentiment polarity of 0.0366. For the concern variable, the COVID-19 attention ratios for politics (7.36%), education (6.61%), and economics (6.57%) were relatively high, followed by health (4.36%) and entertainment (2.19%). The sentiment polarity of political topics (0.0291) was the lowest among these topics, followed by economic (0.1001), health (0.1110), education (0.1184), and entertainment (0.1503) topics.

In general, these data indicate that organizations, as compared to individuals; males, as compared to females; and older people, as compared to young people, are more concerned about the pandemic. In addition, these data indicate that people are more concerned about politics, education, and economics under COVID-19.

#### Bivariate Analysis

**Figure 5:**
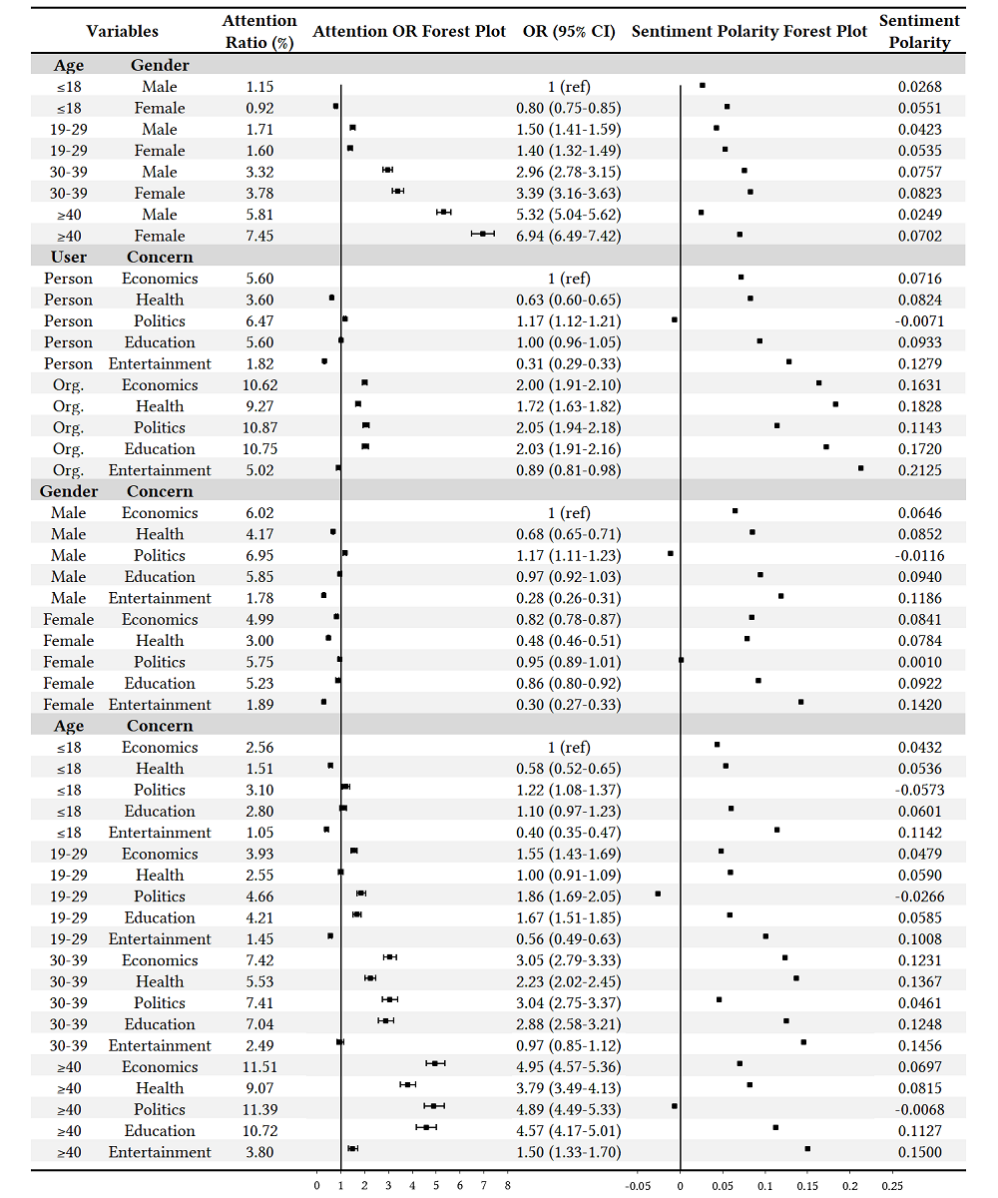
Bivariate analysis of COVID-19 concerns and sentiment polarities among different population groups. OR: odds ratio; Org: organization.

Furthermore, we performed a bivariate analysis on COVID-19 attention and sentiment polarity by crossing any two population characteristic variables, as shown in Figure 5.

It can be seen that many results are consistent with the univariate analysis in the previous section. For example, under the combination of age and gender variables, the attention ratios grew with age, both for males and females. Moreover, females were more positive than males in all age groups. Under the combination of user type and concern variables, the order of concerns for individuals is politics, education, economics, health, and entertainment, which is similar to the univariate results.

However, there are still some noteworthy differences. First, not all females of different ages paid less attention to COVID-19 than males, but as individuals got older, females became more concerned than males, with the highest attention ratio of 7.45% and OR of 6.94 (95% CI 6.49-7.42) in females 40 years or older. Second, males 40 years or older (0.0249) and 18 years or younger (0.0268) were the least positive among all population groups. Third, different from the univariate concern analysis, the order of concerns for groups 30 to 39 years and 40 years or older changed to economics, politics, education, health, and entertainment.

From the bivariate results, we can see that not all the population groups obeyed the same rules, but some of them presented worthy differences under multivariable conditions. We further conducted a deeper exploration in the following trivariate analysis.

#### Trivariate Analysis

**Figure 6:**
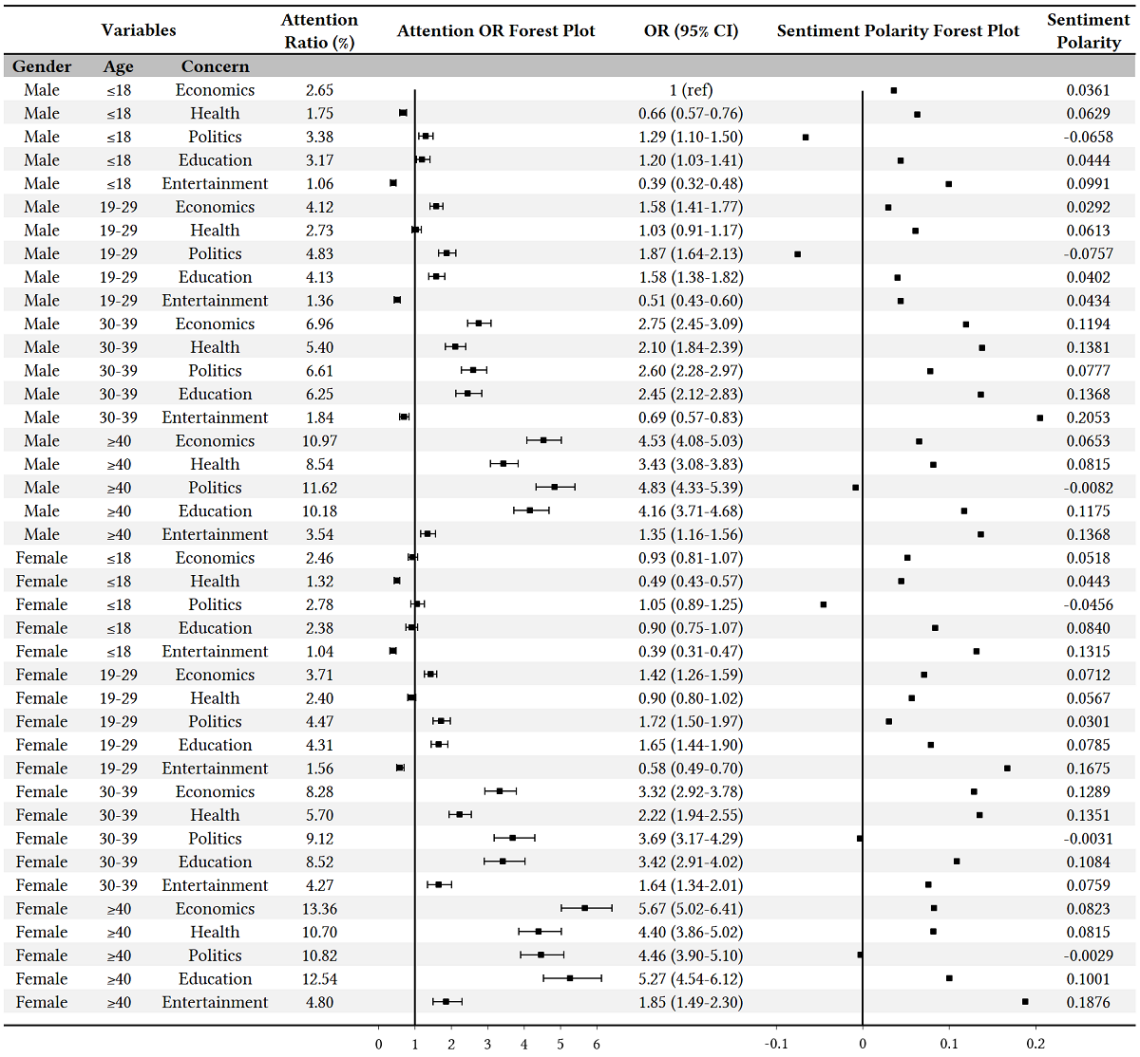
Trivariate analysis of COVID-19 concerns and sentiment polarities among different population groups. OR: odds ratio.

In this part of the study, we crossed the three variables—gender, age, and concern—of population characteristics to study the COVID-19 responses, and a total of 40 combinations were produced, as shown in Figure 6. Since gender and age attributes did not exist in the organization group, this trivariate analysis only concentrated on individuals.

Like in the bivariate analysis, there were some consistent results in the trivariate analysis. For example, the COVID-19 attention ratios increased with age, both for males and females in each topic of concern. Meanwhile, many detailed population differences were also clearly shown in these trivariate results. First, we can see that all of the groups presented different amounts of attention on the five topics of concern. In particular, females 40 years or older paid the greatest amount of attention to economic topics (OR 5.67, 95% CI 5.02-6.41), followed by education topics (OR 5.27, 95% CI 4.54-6.12). As a comparison, males in the same age group (ie, ≥40 years) had the highest concerns regarding political topics (OR 4.83, 95% CI 4.33-5.39), followed by economic (OR 4.53, 95% CI 4.08-5.03) and education (OR 4.16, 95% CI 3.71-4.68) topics. Second, the sentiment polarities of political topics were the lowest in all population groups, of which six had negative values. Lastly, the sentiment polarities of entertainment topics were always the highest among the five topics of concern across all population groups.

### COVID-19 Emotion Analysis

**Figure 7:**
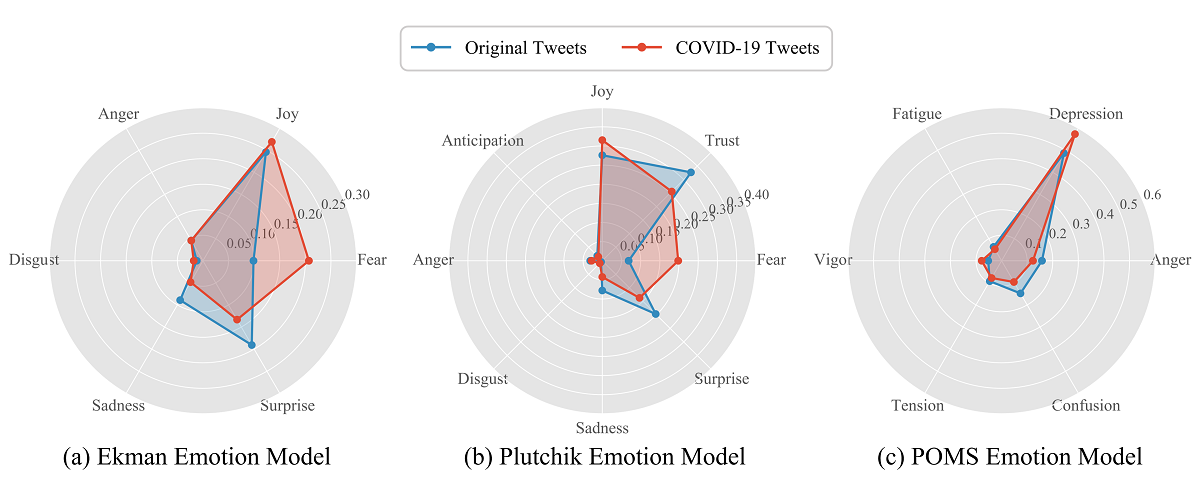
The mean intensity scores for the three emotion models. Scores range from 0 to 1 for each emotion. POMS: Profile of Mood States.

**Figure 8:**
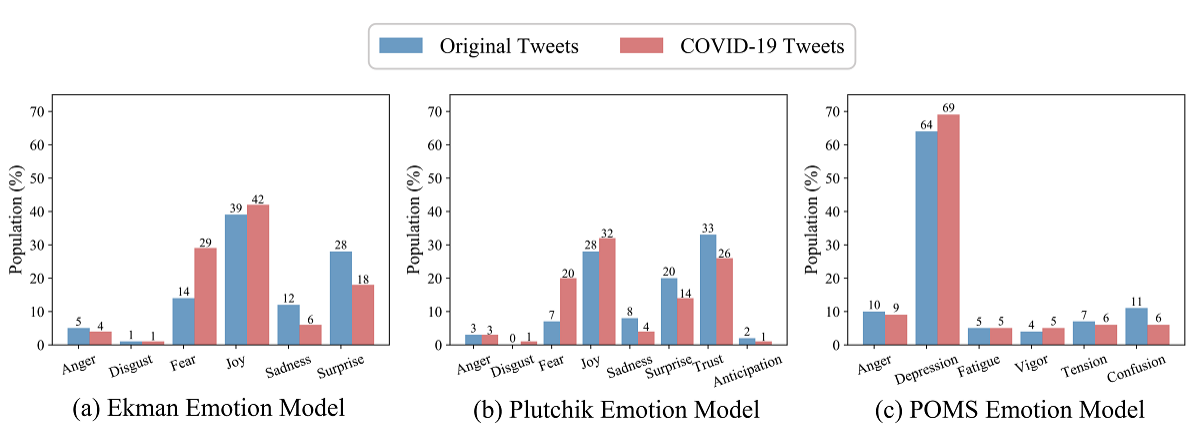
The population distributions of the three emotion models. POMS: Profile of Mood States.

We applied three different emotion models—Ekman’s six basic emotions, Plutchik’s eight primary emotions, and POMS six mood states—to perform emotion detection, both on the original tweets and the COVID-19 tweets. The comparison results are shown in Figures 7 and 8. Figure 7 presents the mean intensity scores of the three emotion models, and Figure 8 shows the population distribution for each emotion from the models based on both original and COVID-19 tweets. As Ekman’s six basic emotions (ie, anger, disgust, fear, joy, sadness, and surprise) are included in Plutchik’s eight emotions, and these six common emotions had the same proportion rank in our experimental results, we only then analyzed Plutchik’s and POMS emotions.

In general, when Plutchik’s emotion model was applied to the original tweets, *trust*, *joy*, and *surprise* were the highest emotions. When the model was applied to COVID-19 tweets, *fear* increased significantly, then *joy*, *trust*, and *fear* became the highest emotions. Meanwhile, when the POMS emotion model was applied to original tweets, *depression* was the most prominent emotion, and when applied to COVID-19 tweets, *depression* became even more prominent.

**Figure 9:**
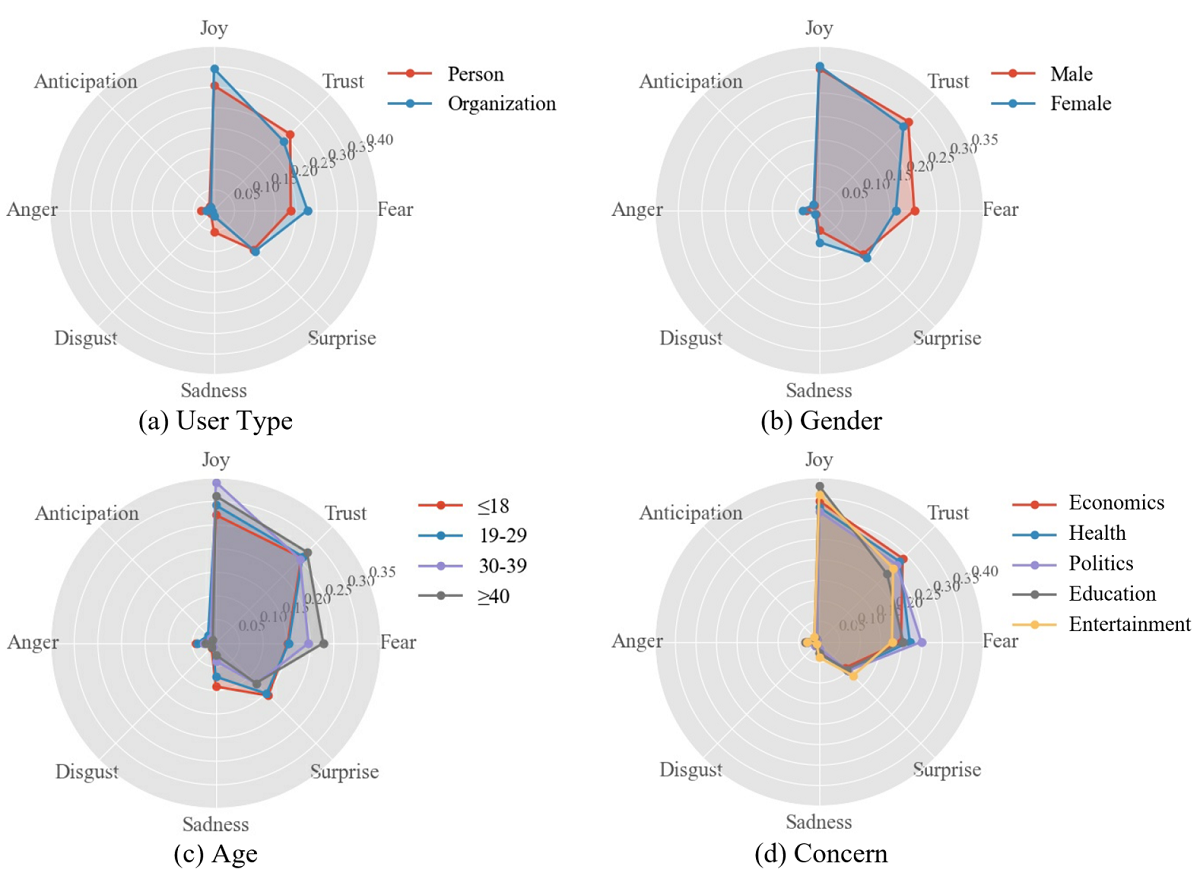
Plutchik emotion analysis on four population characteristics. Scores range from 0 to 1 for each emotion.

**Figure 10:**
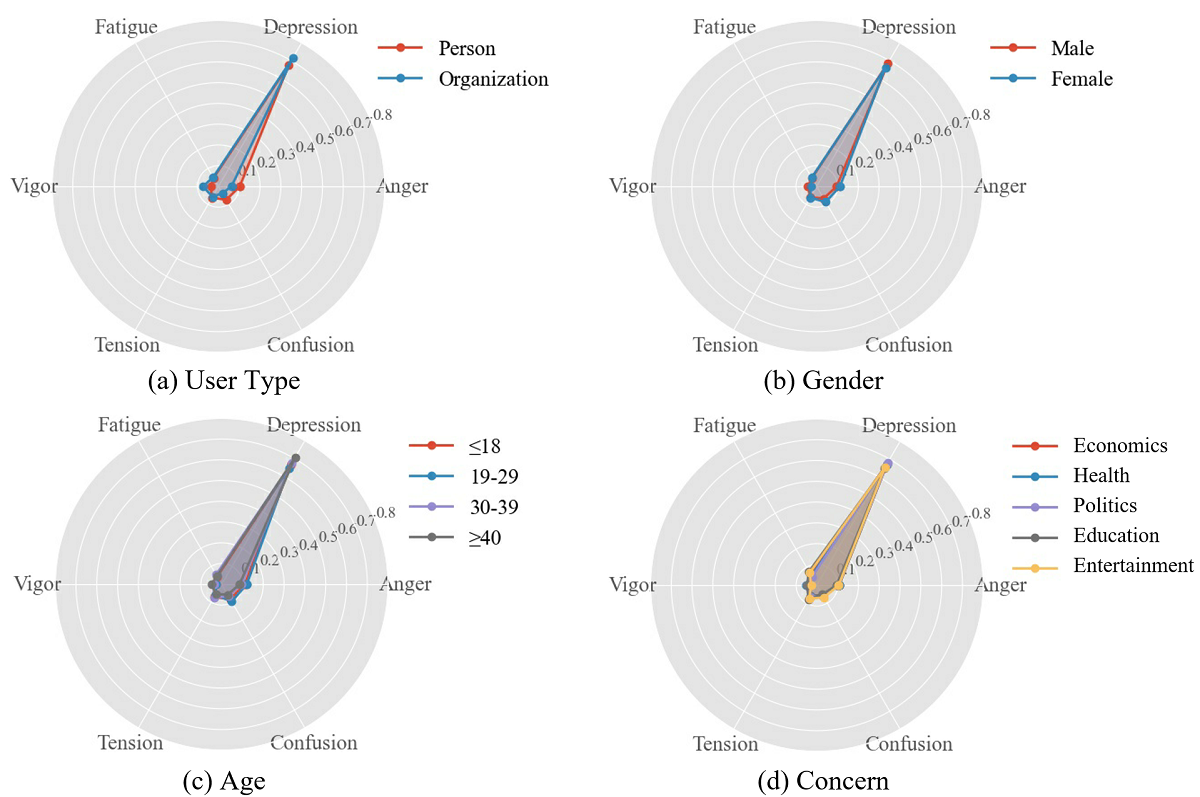
Profile of Mood States (POMS) emotion analysis on four population characteristics. Scores range from 0 to 1 for each emotion.

**Figure 11:**
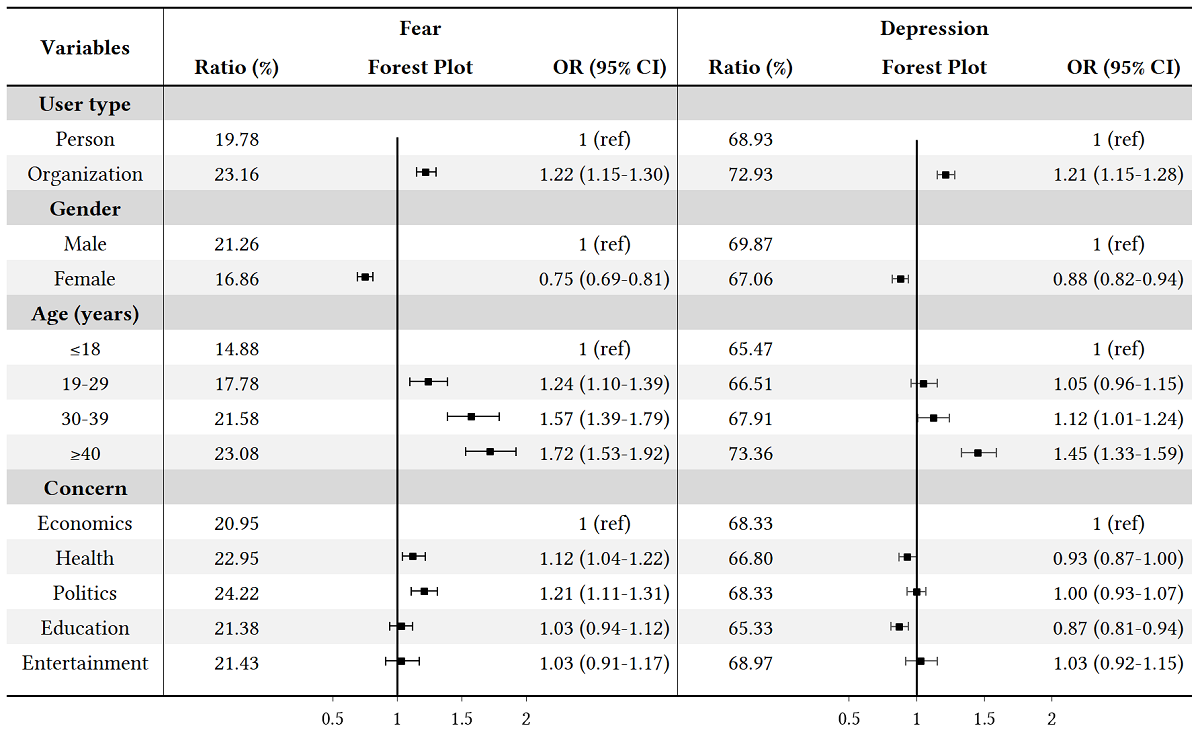
Statistical analysis of emotions related to COVID-19. OR: odds ratio.

Afterward, we studied the differences in emotions considering the population characteristic attributes under COVID-19 by performing a chi-square test on each population attribute for each emotion. The results are shown in Multimedia Appendix 2. Figures 9 and 10 illustrate the emotion analysis by applying Plutchik’s and POMS models to each population characteristic. We observed differences in emotions with respect to population variables, but among all the dominant emotions after applying Plutchik’s and POMS models, *fear* and *depression* had significantly different scores and proportions in different populations. A further detailed statistical analysis was conducted on these two emotions (see Figure 11). We can see that organizations expressed more *fear* and *depression* than individuals, and females expressed less *fear* and *depression* than males. With increasing age, *fear* and *depression* increased significantly; in addition, people expressed more *fear* regarding political and health topics, and more *depression* regarding entertainment, economic, and political topics.

In summary regarding the emotion analysis, it can be concluded that the emotions differed between original tweets and COVID-19 tweets, and they further differed among different population groups during the COVID-19 pandemic.

## Discussion

### Principal Findings

In this study, we analyzed a large amount of Twitter data collected from August 7 to 12, 2020, during the COVID-19 pandemic. In the overall analysis, the average sentiment polarity of COVID-19-related tweets posted by participants was less positive than that of the original tweets. In addition, the population groups under each variable (ie, user type, gender, age, and concern) all showed significant differences (*P*<.001) in response to COVID-19. In univariate analysis, organizations, as compared to individuals; males, as compared to females; and older people, as compared to young people were more concerned about the pandemic and had greater proportions of *fear* and *depression* emotions. In addition, the COVID-19 attention ratios of politics, education, and economics were relatively high, followed by health and entertainment, while the sentiment polarity of politics was the lowest, followed by economics, health, education, and entertainment.

Furthermore, the multivariate analyses showed more fine-grained and meaningful results. Among the findings, it is worth noting that not all female groups paid less attention to COVID-19 than male groups in the same age range, and not all groups’ top concerns were the same. As age increased to above 30 years, females were gradually more concerned about COVID-19 than males. Moreover, females above 40 years of age were the group most concerned about COVID-19, and they were most concerned about economics and education. As a comparison, males in the same age group were most concerned about politics and economics. Males above 40 years of age and below 18 years of age were the least positive in sentiment. Among all the five topics of concern, the sentiment polarities of politics were the lowest in all population groups. These findings demonstrate that there exist population-level disparities in concerns and sentiments about COVID-19 in response to the pandemic during our research period.

We speculate that there are two reasons for the population-level differences. First, they are related to the concrete needs of specific age groups. For example, people older than 30 years of age may pay more attention to COVID-19 impacts on economics, while young people may concentrate more on education. Second, they are also related to the features of this novel coronavirus. Epidemiological studies have shown that the older population is more susceptible to COVID-19 and mortalities among this age group are higher than in other populations [13].

### Limitations

The algorithm of demographic characteristic extraction used in this study is only capable of extracting three basic attributes: user type, gender, and age. Therefore, it is difficult for us to conduct a more detailed multivariable analysis compared with traditional questionnaire methods. In addition, the age range divisions were not fine-grained enough for COVID-19, especially for the group that was 40 years old or above, which covers a wide age range. To support the extraction of more attributes with finer granularity, we plan to optimize the current algorithm or seek new suitable and efficient algorithms for future studies.

### Conclusions

Through large-scale Twitter data mining, this study revealed that salient disparities exist among population groups in terms of their concerns and sentiments regarding COVID-19-related issues. Therefore, it is suggested that government agencies and social organizations should devote specialized attention and support to each population group based on their varied concerns and sentiments experienced during the pandemic. The open source code developed in this study, which was publicly released via GitHub [22], can be easily employed to explore the evolution of population groups regarding their wants, needs, and thoughts during the pandemic for future follow-ups. It can also be repurposed for research and interventions used in combatting other public health emergencies, thanks to the efficient and economic nature of its operation.

## Appendix

Multimedia Appendix 1 Supplemental information about the M3 (multimodal, multilingual, and multi-attribute) model.

Multimedia Appendix 2 Extended details on the data analyses.

## Abbreviations

DenseNet: dense convolutional network
M3: multimodal, multilingual, and multi-attribute
OR: odds ratio
POMS: Profile of Mood States
VADER: Valence Aware Dictionary and Emotional Reasoner
