# Supplementary material for: Understanding Concerns, Sentiments, and Disparities Among Population Groups During the COVID-19 Pandemic Via Twitter Data Mining: Large-scale Cross-sectional Study
Source: J Med Internet Res. 2021 Mar 5;23(3):e26482. doi: 10.2196/26482 (PMC7939057; doi:10.2196/26482)
Supplement: Multimedia Appendix 1 [file jmir_v23i3e26482_app1.docx]

# Multimedia Appendix 1

## Supplemental information about the M3 Model

To evaluate the performance of the M3 model on Twitter data, we first tested the model on three well-known Twitter users, then tested it on a subset of our original English tweets (collected during 7-12 August, 2020) which carries ground truth labels of user type, gender and age explicitly or implicitly. The detailed evaluating processes and results are presented in the following.

First, we select three well-known Twitter users as examples to infer their demographic attributes through the M3 model. It can be seen from Table 1 that the results are significantly accurate.

**Table S1.** Examples of demographic inference on three well-known Twitter users (GT for ground truth, M3 PP for the M3 predicted probability, Per. for person, Org. for organization, M for male, F for female).

| **Name** | **User type** | | | **Gender** | | | **Age (years)** | | | | |
| --- | --- | --- | --- | --- | --- | --- | --- | --- | --- | --- | --- |
|  | **GT** | **M3 PP** | | **GT** | **M3 PP** | | **GT** | **M3 PP** | | | |
|  |  | **Per.** | **Org.** |  | **M** | **F** |  | **<=18** | **19-29** | **30-39** | **>=40** |
| Joseph Robinette Biden Jr. | Per. | 0.9994 | 0.0006 | M | 0.9982 | 0.0018 | 78 | 0.0006 | 0.0002 | 0.0000 | 0.9991 |
| Ivanka Trump | Per. | 1.0000 | 0.0000 | F | 0.0027 | 0.9973 | 39 | 0.0003 | 0.0246 | 0.9504 | 0.0247 |
| BBC News (World) | Org. | 0.1317 | 0.8683 | / | 0.7462 | 0.2538 | / | 0.0048 | 0.0469 | 0.6065 | 0.3419 |

Further, to benchmark the performance of the M3 model on the Twitter dataset we collected, the ground-truth demographic labels of Twitter users are needed to evaluate the predicted results. However, the true attributes of ordinary users are usually too difficult to acquire, especially for age. To overcome this difficulty, we designed an approach to detect Twitter users who have explicitly or implicitly provided demographic information in the tweet texts or profile information. Specifically, the detection procedure is divided into the following four steps. First, we applied a regular pattern with birth keywords (‘born’, ‘years old’, ‘birth’, ‘birthday’) to filter out a subset of Twitter users with possible birthday information. Second, we constructed a gender regular pattern by masculine and feminine words for gender recognition on this subset. Third, we built a regular pattern with organization keywords to distinguish possible organizations. Last, after the above steps, there were still a part of data missing some true attributes, so we completed them manually or deleted them when the truths cannot be determined. We implemented this procedure on our original English dataset (1,015,655), and finally obtained a ground-truth subset of 216 Twitter users, including 45 organizations and 171 individuals (101 males, 70 females).

The benchmark performance of the M3 model on this subset is shown in Table 2. These results demonstrate that the M3 model performs well on our dataset.

**Table S2.** The benchmark performance of the M3 model on the subset of our original English Tweet.

| **Metric** | **User type** | **Gender** | **Age** |
| --- | --- | --- | --- |
| **Macro-F1** | 0.9860 | 0.9572 | 0.7311 |
| **Accuracy** | 99.07% | 95.88% | 77.65% |

For the detailed ground-truth label data and predicted data of these users(according to Twitter policy, only user ids can be provided), see our GitHub repository (https://github.com/cyzhang87/EmulatedQuestionnaireOnTwitter). And, the code for the above procedure can also be found there.
