# Supplementary material for: Understanding Concerns, Sentiments, and Disparities Among Population Groups During the COVID-19 Pandemic Via Twitter Data Mining: Large-scale Cross-sectional Study
Source: J Med Internet Res. 2021 Mar 5;23(3):e26482. doi: 10.2196/26482 (PMC7939057; doi:10.2196/26482)
Supplement: Multimedia Appendix 2 [file jmir_v23i3e26482_app2.docx]

# Multimedia Appendix 2

## Extended Details on Data Analysis

**Table S1.** The chi-square test results of Plutchik emotion model on COVID-19 tweets (ordered by the proportion rank).

| **Emotion** | **Variables** | ***P* value** | **Emotion** | **Variables** | ***P* value** |
| --- | --- | --- | --- | --- | --- |
| Joy | User type | <.001 | Sadness | User type | <.001 |
|  | Gender | .36 |  | Gender | <.001 |
|  | Age | .02 |  | Age | <.001 |
|  | Topic | <.001 |  | Topic | <.001 |
| Trust | User type | .01 | Anger | User type | <.001 |
|  | Gender | .02 |  | Gender | <.001 |
|  | Age | .16 |  | Age | <.001 |
|  | Topic | <.001 |  | Topic | .05 |
| Fear | User type | <.001 | Anticipation | User type | <.001 |
|  | Gender | <.001 |  | Gender | .88 |
|  | Age | <.001 |  | Age | .06 |
|  | Topic | <.001 |  | Topic | <.11 |
| Surprise | User type | .08 | Disgust | User type | <.001 |
|  | Gender | .02 |  | Gender | .60 |
|  | Age | .48 |  | Age | .85 |
|  | Topic | <.001 |  | Topic | .75 |

**Table S2.** The chi-square test results of POMS emotion model on COVID-19 tweets (ordered by the proportion rank).

| **Emotion** | **Variables** | ***P* value** | **Emotion** | **Variables** | ***P* value** |
| --- | --- | --- | --- | --- | --- |
| Depression | User type | <.001 | Tension | User type | <.001 |
|  | Gender | <.001 |  | Gender | 0.17 |
|  | Age | <.001 |  | Age | .01 |
|  | Topic | 0.003 |  | Topic | .05 |
| Anger | User type | <.001 | Fatigue | User type | 0.07 |
|  | Gender | <.001 |  | Gender | 0.20 |
|  | Age | <.001 |  | Age | <.001 |
|  | Topic | 0.27 |  | Topic | .25 |
| Confusion | User type | <.001 | Vigour | User type | <.001 |
|  | Gender | <.001 |  | Gender | <.001 |
|  | Age | <.001 |  | Age | <.001 |
|  | Topic | .71 |  | Topic | .11 |
